# A potential relation between premenstrual symptoms and subjective perception of health and stress among college students: a cross-sectional study

**DOI:** 10.1186/s13030-019-0167-y

**Published:** 2019-10-31

**Authors:** Tamaki Matsumoto, Miho Egawa, Tetsuya Kimura, Tatsuya Hayashi

**Affiliations:** 1 Health Education Course, Department of Education, Faculty of Education, Shitennoji University, 3-2-1 Gakuenmae, Habikino, Osaka 583-8501 Japan; 2 Department of Gynecology and Obstetrics, Kyoto University Graduate School of Medicine, Kyoto, 606-8507 Japan; 3 Graduate School of Human Development and Environment, Kobe University, Kobe, Hyogo 657-8501 Japan; 4 Cognitive and Behavioral Science, Graduate School of Human and Environmental Studies, Kyoto University, Kyoto, 606-8501 Japan

**Keywords:** Premenstrual syndrome, Subjective health, Self-rating stress, Menstrual distress questionnaire, College students

## Abstract

**Background:**

A majority of women from all cultures and socioeconomic levels experience myriad symptoms known as premenstrual syndrome during the days prior to menstruation. The present study investigated commonly reported symptoms in the premenstrual phase among college students. The authors further scrutinized potential factors, including subjective perceptions of health, which may be related to the premenstrual-symptom constellation.

**Methods:**

We conducted a cross-sectional survey, which included 200 participants (mean age: 19.8 ± 0.1 years old). The subjects completed a rating of their premenstrual experiences relative to 46 symptoms in eight categories of the self-reporting menstrual distress questionnaire (MDQ) to evaluate the prevalence and severity of premenstrual symptoms. The participants also answered a standardized health questionnaire regarding subjective perceptions of health, self-rating stress, lifestyle, and demographic variables.

**Results:**

Regardless of severity, the 10 symptoms most often occurring among the participants included skin disorders, irritability, fatigue, mood swings, general aches and pains, lowered school or work performance, backache, painful breasts, weight gain, and swelling. Stepwise multiple regression analysis revealed subjective perception of health (β = 0.28; *p* <  0.001) and self-rating stress (β = 0.18; *p* = 0.008) as the factors most strongly related to the MDQ total scores. In addition, the 19 women who evaluated themselves as “unhealthy and stressed” had greater prevalence of severe or extremely severe physical (general aches and pains) and psychosocial symptoms (confusion, lowered school or work performance, decreased efficiency, loneliness, anxiety, restlessness, mood swings, and depression), compared to the healthy and non-stressed women.

**Conclusions:**

The present study indicates the prevalence of premenstrual symptoms, regardless of severity and number, among college students and suggests that negative subjective perceptions of health and stress may be related to the intensity of premenstrual symptomatology.

## Background

Women of childbearing age have a circumlunar rhythm of the reproductive system. Menstruation, a physiological phenomenon, has multiple biopsychosocial elements, which have repercussions for women from all cultures and socioeconomic levels. In the late luteal phase, for instance, a majority of women experience at least some degree of disharmony of mind and body. This is commonly termed premenstrual syndrome (PMS)—a regular late-luteal recurrence of diverse nonspecific physical, emotional, behavioral, and cognitive symptoms, which usually abates shortly after the onset of menses [1, 2].

More than 200 premenstrual symptoms have been reported, and symptoms and discomfort levels vary from woman to woman [1, 2]. Even when the severity of symptoms does not reach the diagnostic criteria of severe PMS or premenstrual dysphoric disorder (PMDD) [3], the symptomatology could impact an individual’s interpersonal relationships, social interactions, occupational activities, and productivity for her entire reproductive-age life [1, 2]. Especially for young women, premenstrual symptoms can be related to academic performance impairments including poor grades [4] and absenteeism [5]. The symptomatology renders the women more vulnerable to negative health outcomes in later years, such as postpartum depression [6]. After more than half a century of examining the subject, however, research has yet to clarify which symptoms most frequently occur and what types of factors worsen premenstrual complaints, which can start early in the teenage years and commonly occur into the twenties [2, 5].

Subjective health and well-being measurements offer a unique scope with which to capture latent health concerns and conditions that cannot be directly (or cost-effectively) captured through objective measurement [7, 8]. These evaluations are sometimes more reliable predictors of mortality than standard clinical biomarkers [9]. With considerable interest, large-scale population health research has used self-rated health as a good surrogate marker for individual health [10, 11]. Taking these findings into consideration, the authors find it plausible that negatively perceived self-health could associate with undefined biopsychosocial complaints most reproductive-age women experience premenstrually. Limited research, however, has applied such tools to assess women’s mind and body health and/or to explore the etiology of PMS, with its complex web of biopsychosocial factors.

The present study thus aimed to investigate commonly reported symptoms in the premenstrual phase among college students. The authors further scrutinized potential factors, including subjective perceptions of health, which may be related to the premenstrual-symptom constellation.

## Methods

### Subjects

Two hundred twenty-two menstruating women volunteered to participate in a cross-sectional survey. The women, all college students, responded to a campus advertisement. The study protocol was approved in advance by the Institutional Review Board of Shitennoji University and was performed in accordance with the Declaration of Helsinki of the World Medical Association. All subjects received an explanation of the nature and purpose of the study. Before receiving any data about the experiments, all subjects provided written informed consent to participate in the study.

### Measurements

The subjects were asked to complete a standardized health questionnaire, described below, and underwent a brief face-to-face interview [12, 13]. In order to evaluate the prevalence and severity of her premenstrual symptomatology, each subject filled out the self-reporting menstrual distress questionnaire (MDQ) [14]. Briefly, the MDQ consists of 46 symptoms in eight categories: pain, concentration, behavioral change, autonomic reactions, water retention, negative affect, arousal, and control. The subjects rated their experience of all 46 symptoms on the MDQ on a six-point scale ranging from no experience of the symptom to experiencing its most severe level. The total score could, therefore, range from a minimum 46 points to a maximum 276 points.

We assessed subjective perception of health with the question: “What do you think about your current health status?” (“very healthy,” “healthy,” “unhealthy,” or “very unhealthy”). Earlier research revealed that perceived stress has significant effects on increased premenstrual complaints [2, 15–19]. Thus, this study also assessed self-rating of stress among the participants by asking: “How would you rate your current stress level?” (“non-stressful,” “slightly stressful,” “stressful,” or “very stressful”). Referring to previous studies [10, 11], we assigned a dichotomous variable for subjective health (0 if very healthy or healthy; 1 if unhealthy or very unhealthy) and self-rating stress (0 if non-stressful or slightly stressful; 1 if stressful or very stressful) for multiple regression analysis.

Demographic variables consisted of age, body size, medical history, medication, and menstrual cycles in the prior 2 months [12, 13]. None of the subjects had been clinically diagnosed with gynecological problems, such as amenorrhea, dysmenorrhea and endometriosis. None of the women reported taking oral contraceptives to control the menstrual cycle. No subjects suffered from psychiatric diseases. None of the subjects had been clinically diagnosed with diabetes mellitus, hypertension, hyperlipidemia, or other lifestyle-related diseases that could affect the degree of subjective health and quality of life [8]. As to medical history, nine participants reported that they had experienced the following acute diseases, including appendicitis, candidal vaginitis, otitis media, cystitis, iron-deficiency anemia, or food poisoning, which had been healed at the time this cross-sectional survey was conducted. Body mass index (BMI) was calculated as body weight divided by height squared in meters. As to lifestyle habits, we asked the subjects: “Do you eat breakfast every morning?” and “Do you regularly exercise (more than once per week)?” [5]. As we mentioned above, we dichotomized the responses, yes (0) or no (1), for multiple regression analysis. To measure general sleep duration the participants answered the question, “How many hours do you sleep?” [10].

### Statistical analysis

All descriptive and inferential statistical analyses were performed using a commercial software package (IBM SPSS Statistics Version 25; IBM Corp., Armonk, NY, USA). Internal consistency of the MDQ was evaluated by calculating Chronbach’s alpha coefficients. Multiple regression analysis with stepwise selection was performed to examine factors potentially related to premenstrual symptoms. The effects of subjective health and self-rating stress and their interaction were evaluated using two-way analysis of variance (ANOVA) to investigate the influence of these two factors on total and sub-scores of the MDQ. Pearson’s chi-square test, Fisher’s exact test, and unpaired t-test were performed to compare the prevalence of premenstrual symptoms, lifestyle factors, and demographic variables between two groups—“healthy and non-stressed” and “unhealthy and stressed” groups. Values are reported as means ± standard deviations. Statistical tests were two-sided and *p* <  0.05 was adopted as the level of significance.

## Results

A total of 222 students willingly assented to participate in the study, but data from 22 participants were excluded because of missing information on demographic variables, lifestyle factors, or the MDQ. Consequently, we analyzed the data of 200 college students aged 18–25 years. Table 1 shows the background characteristics of the 200 participants.

**Table 1:** Background characteristics of the participants (*N* = 200)

Variables	
Age (yrs)	19.8 ± 1.0
Height (cm)	158.5 ± 5.3
Weight (kg)	51.4 ± 6.0
Body Mass Index (kg/m^2^)	20.5 ± 2.1
Menstrual cycle (days)	30.0 ± 4.7
Sleeping durations (time)	6.7 ± 1.1
Breakfast eating habits, no. (%)	150 (75.0)
Regular exercise habits, no. (%)	55 (27.5)

Values given as means ± standard deviation

In this study, the Chronbach’s alpha coefficient of the MDQ was 0.94. The values for the eight subcategories were as follows: pain 0.70, concentration 0.87, behavioral change 0.88, autonomic reactions 0.69, water retention 0.72, negative affect 0.93, arousal 0.80, and control 0.64. The MDQ total score varied among subjects, from 46 to 171. This indicates that, with one exception, 199 students (99.5%) experienced at least one symptom in the premenstrual phase. The prevalence of each premenstrual symptom of the MDQ is shown in Table 2. Regardless of the severity, more than half of the participants had 19 symptoms (*) subcategorized in four factors: pain, behavioral change, water retention and negative affect. The ten symptoms (*1–10) most often occurring among the participants include: skin disorders, irritability, fatigue, mood swings, general aches and pains, lowered school or work performance, backache, painful breasts, weight gain, and swelling. As Table 3 shows, 122 participants (61.0%) experienced at least one “severe” or “extremely severe” symptom in the premenstrual phase. Among them, 16 students (8.0%) had more than 11 severe to extremely severe symptoms. It should be noted that, according to face-to-face interviews, the general health questionnaire, and total scores of the MDQ, the severity of symptoms the participants experienced did not cause serious disturbance to daily activities or quality of life. In addition, none of the participants sought urgent clinical treatment to ameliorate premenstrual symptomatology.

**Table 2:** Prevalence rates of premenstrual symptoms (*N*=200)

		Severity					
Factors	Symptoms	No experience	Very mild	Mild	Moderate	Severe	Extremely severe
Pain	^*^Muscle Stiffness	92 (46.0)	38 (19.0)	30 (15.0)	22 (11.0)	16 (8.0)	2 (1.0)
	^*5^General aches and pains	53 (26.5)	31 (15.5)	41 (20.5)	43 (21.5)	25 (12.5)	7 (3.5)
	Headache	112 (56.0)	39 (19.5)	24 (12.0)	16 (8.0)	7 (3.5)	2 (1.0)
	^*7^Backache	65 (32.5)	40 (20.0)	35 (17.5)	37 (18.5)	16 (8.0)	7 (3.5)
	^*3^Fatigue	37 (18.5)	47 (23.5)	46 (23.0)	43 (21.5)	24 (12.0)	3 (1.5)
	Cramps	196 (98.0)	3 (1.5)	0 (0)	1 (0.5)	0 (0)	0 (0)
Concentration	Insomnia	140 (70.0)	27 (13.5)	19 (9.5)	7 (3.5)	6 (3.0)	1 (0.5)
	Forgetfulness	141 (70.5)	32 (16.0)	18 (9.0)	6 (3.0)	3 (1.5)	0 (0)
	Confusion	139 (69.5)	24 (12.0)	23 (11.5)	8 (4.0)	6 (3.0)	0 (0)
	Lowered judgment	105 (52.5)	37 (18.5)	31 (15.5)	17 (8.5)	9 (4.5)	1 (0.5)
	^*^Difficulty concentrating	91 (45.5)	39 (19.5)	35 (17.5)	19 (9.5)	15 (7.5)	1 (0.5)
	^*^Distractible	94 (47.0)	43 (21.5)	30 (15.0)	20 (10.0)	11 (5.5)	2 (1.0)
	Accidents	178 (89.0)	12 (6.0)	6 (3.0)	2 (1.0)	1 (0.5)	1 (0.5)
	Lowered motor coordination	138 (69.0)	31 (15.5)	16 (8.0)	9 (4.5)	5 (2.5)	1 (0.5)
Behavioral change	^*6^Lowered school or work performance	53 (26.5)	56 (28.0)	41 (20.5)	32 (16.0)	15 (7.5)	3 (1.5)
	^*^Take naps; stay in bed	95 (47.5)	30 (15.0)	28 (14.0)	23 (11.5)	19 (9.5)	5 (2.5)
	^*^Stay at home	100 (50.0)	41 (20.5)	28 (14.0)	16 (8.0)	11 (5.5)	4 (2.0)
	Avoid social activities	113 (56.5)	38 (19.0)	25 (12.5)	11 (5.5)	11 (5.5)	2 (1.0)
	^*^Decreased efficiency	92 (46.0)	46 (23.0)	33 (16.5)	20 (10.0)	9 (4.5)	0 (0)
Autonomic reactions	Dizziness, faintness	139 (69.5)	27 (13.5)	22 (11.0)	6 (3.0)	5 (2.5)	1 (0.5)
	Cold sweats	159 (79.5)	20 (10.0)	11 (5.5)	6 (3.0)	3 (1.5)	1 (0.5)
	Nausea, vomiting	146 (73.0)	24 (12.0)	20 (10.0)	4 (2.0)	3 (1.5)	3 (1.5)
	Hot flashes	176 (88.0)	13 (6.5)	7 (3.5)	3 (1.5)	0 (0)	1 (0.5)
Water retention	^*9^Weight gain	69 (34.5)	38 (19.0)	43 (21.5)	35 (17.5)	12 (6.0)	3 (1.5)
	^*1^Skin disorders	26 (13.0)	34 (17.0)	50 (25.0)	41 (20.5)	38 (19.0)	11 (5.5)
	^*8^Painful breasts	68 (34.0)	30 (15.0)	38 (19.0)	35 (17.5)	23 (11.5)	6 (3.0)
	^*10^Swelling	77 (38.5)	41 (20.5)	45 (22.5)	17 (8.5)	14 (7.0)	6 (3.0)
Negative affect	Crying	122 (61.0)	27 (13.5)	16 (8.0)	18 (9.0)	14 (7.0)	3 (1.5)
	Loneliness	117 (58.5)	27 (13.5)	22 (11.0)	13 (6.5)	20 (10.0)	1 (0.5)
	^*^Anxiety	90 (45.0)	38 (19.0)	26 (13.0)	22 (11.0)	22 (11.0)	2 (1.0)
	^*^Restlessness	78 (39.0)	48 (24.0)	33 (16.5)	24 (12.0)	17 (8.5)	0 (0)
	^*2^Irritability	34 (17.0)	34 (17.0)	52 (26.0)	39 (19.5)	33 (16.5)	8 (4.0)
	^*4^Mood swings	45 (22.5)	34 (17.0)	49 (24.5)	32 (16.0)	30 (15.0)	10 (5.0)
	^*^Depression	100 (50.0)	28 (14.0)	26 (13.0)	24 (12.0)	21 (10.5)	1 (0.5)
	Tension	142 (71.0)	31 (15.5)	12 (6.0)	9 (4.5)	5 (2.5)	1 (0.5)
Arousal	Affectionate	143 (71.5)	21 (10.5)	23 (11.5)	10 (5.0)	3 (1.5)	0 (0)
	Orderliness	146 (73.0)	27 (13.5)	13 (6.5)	6 (3.0)	4 (2.0)	4 (2.0)
	Excitement	147 (73.5)	28 (14.0)	15 (7.5)	5 (2.5)	5 (2.5)	0 (0)
	Feeling of well-being	168 (84.0)	17 (8.5)	11 (5.5)	3 (1.5)	1 (0.5)	0 (0)
	Bursts of energy, activity	167 (83.5)	17 (8.5)	8 (4.0)	6 (3.0)	2 (1.0)	0 (0)
Control	Feeling of suffocation	168 (84.0)	17 (8.5)	9 (4.5)	3 (1.5)	3 (1.5)	0 (0)
	Chest pains	155 (77.5)	25 (12.5)	12 (6.0)	6 (3.0)	2 (1.0)	0 (0)
	Ringing in the ears	180 (90.0)	11 (5.5)	7 (3.5)	1 (0.5)	1 (0.5)	0 (0)
	Heart pounding	172 (86.0)	13 (6.5)	9 (4.5)	6 (3.0)	0 (0)	0 (0)
	Numbness, tingling	190 (95.0)	5 (2.5)	1 (0.5)	2 (1.0)	2 (1.0)	0 (0)
	Blind spots, fuzzy vision	166 (83.0)	17 (8.5)	12 (6.0)	3 (1.5)	1 (0.5)	1 (0.5)

Values given as numbers (percentage)

^*^Symptoms experienced by more than half of the participants

^*1-10^Ten symptoms most frequently experienced by the participants

**Table 3:** Participants experiencing severe to extremely severe symptoms

Severe to extremely severe symptoms	Subjects
None	78 (39.0)
1–5 symptoms	79 (39.5)
6–10 symptoms	27 (13.5)
11–20 symptoms	16 (8.0)

Values are given as numbers (percentage)

**Fig. 1:**
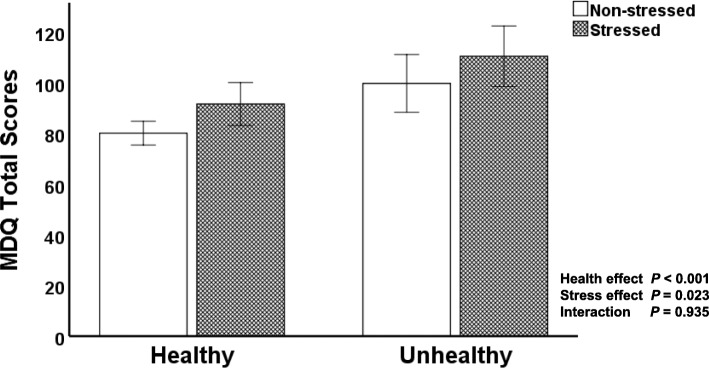
Comparison of the MDQ total scores among four groups categorized by subjective perception of health and stress

We performed multiple linear regression analysis with stepwise selection to determine how well the combination of the seven independent variables (age, BMI, subjective perception of health, self-rating stress, regular exercise habits, breakfast eating habits, and sleep duration) explains the variance in the MDQ total scores. We should mention that, referring to previous studies [2, 5, 15–25], these seven variables were selected as potential factors that may be related to the premenstrual-symptom constellation. As Table 4 shows, multivariable analysis revealed subjective perception of health (β = 0.28; *p* < 0.001) and self-rating stress (β = 0.18; *p* = 0.008) as the factors most strongly related to the severity of premenstrual symptoms evaluated by the MDQ total scores. Two-way ANOVA demonstrated that subjective perceptions of health and self-rating stress had significant effects on the MDQ total scores (health effect: *F*[1, 196] = 15.7, *p* < 0.001; stress effect: *F*[1, 196] = 5.2, *p* = 0.023) (Fig. 1). The authors found no significant interaction of subjective health and stress on the MDQ total scores (health x stress effect: *F*[1, 196] = 0.007, *p* = 0.935). As to the sub-categories of the MDQ, the sub-scores of three factors—pain, concentration, and negative affect—significantly increased in unhealthy and stressful situations (Table 5). Subjective perception of health, not self-rating stress, had a significant effect on four factors: behavioral change, autonomic reactions, arousal, and control. In contrast, neither subjective health nor stress status had any statistically significant effect on the factor of water retention.

**Table 4:** Results of stepwise multiple regression analysis: Independent variables influencing MDQ total scores

Independent variables	B	95%CI	*β*	*P* value
Subjective health	19.3	9.92–28.7	0.28	< 0.001**
Self-rating stress	11.3	2.95–19.6	0.18	0.008**

B: un-standardized partial regression coefficients; *β*: standard partial regression coefficient; *CI* Confidence interval

ANOVA *F* = 14.7 (2, 197), *P* < 0.001

*R*^2^ = 0.13

**P < 0.01

**Table 5:** Effects of subjective perception of health and stress on MDQ subscores

	Healthy		Unhealthy		ANOVA
	Non-stressful (*n* = 122)	Stressful (*n* = 38)	Non-stressful (*n* = 21)	Stressful (*n* = 19)	
Pain	12.7 ± 4.9	13.6 ± 3.4	14.1 ± 4.8	17.4 ± 4.9	Health effect: *F*(1,196) = 9.10, *p* = 0.003**
					Stress effect: *F*(1,196) = 5.97, *p* = 0.015*
					Interaction: *F*(1,196) = 1.93, *p* = 0.166
Concentration	12.0 ± 5.5	14.8 ± 6.7	15.8 ± 7.5	18.5 ± 6.0	Health effect: *F*(1,196) = 11.32, *p* = 0.001**
					Stress effect: *F*(1,196) = 6.17, *p* = 0.014*
					Interaction: *F*(1,196) < 0.001, *p* = 0.990
Behavioral change	10.0 ± 5.1	10.7 ± 4.7	12.3 ± 6.8	14.3 ± 6.3	Health effect: *F*(1,196) = 9.30, *p* = 0.003**
					Stress effect: *F*(1,196) = 1.80, *p* = 0.181
					Interaction: *F*(1,196) = 0.46, *p* = 0.498
Autonomic reactions	5.2 ± 2.0	5.6 ± 2.7	7.3 ± 4.1	6.9 ± 3.1	Health effect: *F*(1,196) = 12.86, *p* = < 0.001**
					Stress effect: *F*(1,196) = 0.003, *p* = 0.958
					Interaction: *F*(1,196) = 0.61, *p* = 0.436
Water retention	10.3 ± 4.2	11.2 ± 4.0	12.4 ± 4.7	11.5 ± 3.9	Health effect: *F*(1,196) = 2.59, *p* = 0.109
					Stress effect: *F*(1,196) = 0.003, *p* = 0.954
					Interaction: *F*(1,196) = 1.52, *p* = 0.220
Negative affect	16.2 ± 8.0	20.8 ± 9.5	20.6 ± 9.6	24.5 ± 9.5	Health effect: *F*(1,196) = 6.53, *p* = 0.011*
					Stress effect: *F*(1,196) = 7.26, *p* = 0.008**
					Interaction: *F*(1,196) = 0.07, *p* = 0.791
Arousal	6.6 ± 3.1	7.3 ± 3.2	8.3 ± 4.0	8.6 ± 4.0	Health effect: *F*(1,196) = 5.95, *p* = 0.016*
					Stress effect: *F*(1,196) = 0.70, *p* = 0.404
					Interaction: *F*(1,196) = 0.12, *p* = 0.726
Control	7.0 ± 2.0	7.5 ± 2.2	8.9 ± 3.4	8.7 ± 3.5	Health effect: *F*(1,196) = 12.54, *p* = < 0.001**
					Stress effect: *F*(1,196) = 0.15, *p* = 0.701
					Interaction: *F*(1,196) = 0.43, *p* = 0.511

Vales given as means ± standard deviation

Statistical significance, **p < 0.01, *p < 0.05

We further scrutinized the effects of negative subjective perception of health and stress on premenstrual severity together with lifestyle factors and demographic variables among the college students. As to the prevalence of premenstrual symptoms, the 19 women who evaluated themselves as “unhealthy and stressed” had greater prevalence of severe or extremely severe physical (general aches and pains) and psychosocial symptoms (confusion, lowered school or work performance, decreased efficiency, loneliness, anxiety, restlessness, mood swings, and depression) on the MDQ scale, compared to the 122 healthy and non-stressed women. The statistical analysis revealed no significant difference in the prevalence of the other 37 symptoms between the two groups. Among background and lifestyle risk factors, the percentage of breakfast eating habits was significantly lower in the “unhealthy and stressed” group than in the “healthy and non-stressed” group (*p* = 0.048). We found no significant difference in other lifestyle factors or demographic variables (Table 6).

**Table 6:** Comparison of ‘healthy & non-stressed’ and ‘unhealthy & stressed’ groups

	Healthy & Non-stressed (*n* = 122)	Unhealthy & Stressed (*n* = 19)	*P*-value
Background and lifestyle risk factors, no. (%)			
Age (yrs)	19.7 ± 0.9	19.9 ± 0.8	0.402
Body Mass Index (kg/m^2^)	20.4 ± 2.1	19.5 ± 2.0	0.178
Menstrual cycle (days)	30.0 ± 3.8	29.0 ± 3.5	0.265
Sleeping durations (time)	6.7 ± 1.0	6.6 ± 1.6	0.505
Breakfast eating habits, no. (%)	91 (74.6)	10 (52.6)	0.048*
Regular exercise habits, no. (%)	32 (26.2)	4 (21.1)	0.630
Premenstrual symptoms (over severe severity), no. (%)			
General aches and pains	19 (15.6)	7 (36.8)	0.026*
Confusion	0 (0)	3 (15.8)	0.002**
Lowered school or work performance	8 (6.6)	4 (21.1)	0.035*
Decreased efficiency	1 (0.8)	3 (15.8)	0.008**
Loneliness	8 (6.6)	5 (26.3)	0.006**
Anxiety	8 (6.6)	6 (31.6)	0.001**
Restlessness	7 (5.7)	6 (31.6)	< 0.001**
Mood swings	17 (13.9)	8 (42.1)	0.003**
Depression	10 (8.2)	5 (26.3)	0.017*

Vales given as means ± standard deviation

Statistical significance, **p < 0.01, *p < 0.05

## Discussion

A number of population-based epidemiological investigations on the prevalence of premenstrual complications have been conducted worldwide. Although research designs and methods differ among the studies, and most of them are based on retrospective rather than prospective recording, the findings have been reasonably congruent. They indicate that nearly 90% of women of reproductive age experience at least one cyclical premenstrual symptom [2, 4, 19, 26, 27]. The present study demonstrated that, with one exception, 199 students (99.5%) experienced at least one symptom listed on the MDQ premenstrually. Regardless of severity, the ten symptoms most often occurring among the participants include: skin disorders, irritability, fatigue, mood swings, general aches and pains, lowered school or work performance, backache, painful breasts, weight gain, and swelling. The results of our study agree with those reported in earlier studies, which indicate that regardless of ethnicity, women in their late teens and early twenties frequently experience such premenstrual complications [4, 19, 21, 24, 26, 28]. As to the severity, epidemiological surveys found a certain percentage (7.7–26.6%) of college students suffering from PMDD—a particularly severe form of PMS defined as a distinct premenstrual affective disorder [29, 30]. In this study, 122 students (61%) experienced at least one severe or extremely severe symptom in the premenstrual phase. Since none of them mentioned that their premenstrual symptoms disturbed academic performance, normal social activities, or relationships, we assume that their severity did not reach the level of severe PMS or PMDD. Although we need further investigation with prospective recordings to precisely evaluate premenstrual conditions, the study in hand reconfirms that a majority of college students are commonly aware of mind and body disharmony with a wide range of severity, in the late luteal phase as previous studies have presented [4, 21, 28–30].

The etiopathogenesis of the complex web of biopsychosocial factors of premenstrual symptomatology remains enigmatic. The present study demonstrates that negative subjective perception of health is significantly related to premenstrual symptomatic features, including its prevalence, type, and severity, in college students. As substantial evidence from earlier PMS research has shown [2, 15–19], this study also clarified that self-rating stress strongly relates to premenstrual symptomatology as assessed by MDQ scores. We further revealed that the 19 women who evaluated themselves as “unhealthy and stressed” had greater prevalence of severe or extremely severe premenstrual complaints consisting of general aches and pains, confusion, lowered school or work performance, decreased efficiency, loneliness, anxiety, restlessness, mood swings, and depression. The results, in other words, indicate that the students who felt unhealthy and stressed had more psychosocial and behavioral symptoms than physical ones in the premenstrual phase.

Subjective health and global self-ratings of health have been identified as a critical indicators of the multi-dimensional construct, health [8, 9]. Poorly perceived health links various adverse psychosocial states such as social isolation, negative life events, depression, and job stress [9]. In addition, self-evaluations of health were related to personal health practices, such as dietary behaviors, physical activity, sleeping, and smoking habits [10, 11, 31]. Such tools have been rarely used to explore premenstrual features; however, we found a 2016 Korean study that revealed women premenstrually experiencing moderate to severe levels of negative affect or intense symptoms of behavioral change had significantly lower scores of perceived health status and quality of life, compared to women with mild premenstrual symptoms [20]. In contrast to subjective health, an association between self-rating stress and premenstrual symptoms has been apparent. For instance, a cross-sectional study with 448 students recruited from three universities in Pakistan demonstrated that 81.5% of the students reported stress exacerbated their premenstrual symptoms [19]. While supporting the findings obtained from cross-sectional studies [15, 16, 18, 19], a longitudinal study in the US [17] elucidated that women with high stress in the previous month were significantly more likely to report an increased number and severity of symptoms in subsequent perimenstrual (premenstrual and menstrual) phases. In addition, changing stress levels across the two cycles were associated with a changing pattern of symptom severity. Methodologies for measuring premenstrual symptoms, perceived health, and self-rating stress are not always consistent among researchers. Taking previous findings [15–20] into consideration, together with the outcomes from the present study, however, we could interpret that negative subjective perception of health along with high self-rating of stress might, at least in part, be related to worsening premenstrual health conditions among reproductive-age women.

In addition to the significant effects of perceived health and stress on premenstrual symptoms, the present study found that the percentage of breakfast eating habits was significantly lower in the “unhealthy and stressed” group than in the “healthy and non-stressed” group. The result is consistent with previous investigations with female college students: A questionnaire survey conducted in two colleges in Japan found a significantly higher population with a self-perception of poor general health among the group that skipped breakfast [23]. The survey also indicated that skipping breakfast adversely affects menstrual disorders in college students. According to a 2016 Turkish epidemiological research, college students with unhealthy behaviors, including irregular breakfast habits, had higher PMS scores [24]. The mechanism through which breakfast contributes to improving premenstrual symptoms remains unclear. Ferrer-Cascales et al. [32] suggested, however, that consuming carbohydrates at breakfast could boost beneficial nutrients for the brain after night fasting as it reduces levels of cortisol production and thereby decreases stress signals. Conversion of carbohydrates into glucose is essential for the formation of tryptophan, a precursor protein for the synthesis of serotonin, which regulates depressive symptoms, irritable mood, and cognitive functioning—all representative of the symptom-complex in the premenstrual phase.

As a first-line therapy, lifestyle modification is recommended for all women experiencing premenstrual symptoms [2]. In addition, health education programs on the effects of ovarian hormones and menstrual cycles on biopsychosocial aspects could be helpful for college students to increase the predictability of menstruation-related problems [2, 5]. A series of the authors’ investigations revealed a significant late-luteal increase in sympathetic nerve activity and decrease in parasympathetic nerve activity [12, 33]. Holistic healing treatments however, improved such autonomic imbalance [13, 34]. Taken together, the present study further implies that women in the early reproductive-age stage should learn about such menstrual-cyclic mind and body fluctuations, acquire strategies for managing stress, and conduct healthy behaviors, which could ameliorate premenstrual symptoms and, ultimately, improve quality of life.

Although the present study entails an important advance in comprehending premenstrual features in college students, we should address some limitations. First, the cross-sectional design of the study did not allow us to establish causal relationships between the variables studied. Second, the retrospective type of questionnaire could result in an overestimation of the prevalence of PMS by the participants. Prospective recording of menstrual cycle-related symptoms at least for 2 months is needed to detect frequently occurring symptoms premenstrually. Third, lifestyle factors including breakfast eating habits, regular exercise habits, and sleeping duration, were also based on self-reporting, which may be subject to error. Finally, the present study included a small, selective, and unevenly distributed sample size. This could limit the outcomes of our study to generalizability.

## Conclusions

The present study indicates the prevalence of premenstrual symptoms, regardless of severity and number, among college students and suggests that negative subjective perceptions of health and stress may be related to the intensity of the premenstrual symptoms a majority of women experience. In addition, a lower quality of lifestyle, which might include skipping breakfast, for example, would adversely influence such late-luteal symptomatology. With the trend toward younger menarche and a lower birth rate, women spend a greater proportion of their lives menstruating. The findings, thus, further imply the need for developing preventive health education programs for managing stress and improving the subjective health of women in the early reproductive-age stage.

## Data Availability

Data cannot be shared publicly because datasets have ethical or legal restrictions for public deposition owing to inclusion of sensitive information from the human participants. All inquiries should be addressed to the corresponding author.

## Abbreviations

ANOVA: Analysis of variance
BMI: Body mass index
MDQ: Menstrual distress questionnaire
PMDD: Premenstrual dysphoric disorder
PMS: Premenstrual syndrome
